# Multiplex detection in tonsillar tissue of all known human polyomaviruses

**DOI:** 10.1186/s12879-017-2479-5

**Published:** 2017-06-08

**Authors:** Mohammadreza Sadeghi, Yilin Wang, Torbjörn Ramqvist, Leena-Maija Aaltonen, Lari Pyöriä, Mari Toppinen, Maria Söderlund-Venermo, Klaus Hedman

**Affiliations:** 1 Virology, University of Helsinki, Helsinki, Finland; 2 Department of Oncology-Pathology, Karolinska Institutet, Stockholm, Sweden; 3 Department of Otorhinolaryngology-Head and Neck Surgery, Helsinki University Hospital, Helsinki, Finland; 4 University of Helsinki, Helsinki, Finland; 5 Helsinki University Hospital, HUSLAB, Helsinki, Finland

**Keywords:** HPyV, PCR, Luminex, Tonsil

## Abstract

**Background:**

In the past few years, eleven new human viruses have joined the two previously known members JCPyV and BKPyV of the *Polyomaviridae* family, by virtue of molecular methods. Serology data suggest that infections with human polyomaviruses (HPyVs) occur since childhood and the viruses are widespread in the general population. However, the viral persistence sites and transmission routes are by and large unknown. Our previous studies demonstrated that the four new HPyVs – KIPyV, WUPyV, MCPyV and TSPyV – were present in the tonsils, and suggested lymphoid tissue as a persistent site of these emerging human viruses.

We developed a Luminex-based multiplex assay for simultaneous detection of all 13 HPyVs known, and explored their occurrence in tonsillar tissues of children and adults mostly with tonsillitis or tonsillar hypertrophy.

**Methods:**

We set up and validated a new Luminex-based multiplex assay by using primer pairs and probes targeting the respective HPyV viral protein 1 (VP1) genes. With this assay we tested 78 tonsillar tissues for DNAs of 13 HPyVs.

**Results:**

The multiplex assay allowed for simultaneous detection of 13 HPyVs with high analytical sensitivity and specificity, with detection limits of 10^0^–10^2^ copies per microliter, and identified correctly all 13 target sequences with no cross reactions. HPyV DNA altogether was found in 14 (17.9%) of 78 tonsils. The most prevalent HPyVs were HPyV6 (7.7%), TSPyV (3.8%) and WUPyV (3.8%). Mixed infection of two HPyVs occurred in one sample.

**Conclusions:**

The Luminex-based HPyV multiplex assay appears highly suitable for clinical diagnostic purposes and large-scale epidemiological studies. Additional evidence was acquired that the lymphoid system plays a role in HPyV infection and persistence. Thereby, shedding from this site during reactivation might take part in transmission of the newly found HPyVs.

## Background

Thirteen human polyomaviruses (HPyVs) have been identified to date. Up to 2007, only two HPyVs had been introduced by cell culture: BK polyomavirus (BKPyV) and JC polyomavirus (JCPyV), coincidentally in 1971 [1, 2]. Since then, eleven HPyVs have been discovered by modern molecular techniques [3–14]. All HPyVs have high seroprevalences in human populations ranging from 40 to 90% in adults [15–26]. Typically, the primary infections occur asymptomatically during childhood and are followed by lifelong DNA persistence. In immunocompromised hosts some of these viruses give rise to severe disorders such as nephropathy, progressive multifocal leukoencephalopathy, Merkel cell carcinoma or trichodysplasia spinulosa [5, 7, 27, 28]. The other HPyVs, i.e. KIPyV, WUPyV, HPyV6, HPyV7, HPyV9, HPyV10 (MWPyV), Saint Louis polyomavirus (STLPyV), HPyV12 and New Jersey polyomavirus (NJPyV)-2013, have up to date not been definitively associated with specific diseases.

On the other hand, evidence suggests that tonsils are permissive for JCPyV and BKPyV, indicating that this tissue might play a role in the viral persistence [29–34]. KIPyV and WUPyV, the first two novel polyomaviruses discovered in the respiratory secretions of children with acute respiratory symptoms, [3, 4] might also remain in lymphoid tissue as suggested by detection of the corresponding sequences in tonsils [29, 35, 36]. We have reported the occurrence of DNA of MCPyV and TSPyV in tonsillar tissue, suggesting persistence in lymphoid tissue or mucosa [36, 37]. MCPyV is a common skin commensal and causes about 80% of cases of Merkel cell carcinoma (MCC) [5, 38–40]. TSPyV induces the rare skin disease trichodysplasia spinulosa (TS) in immunocompromised patients [7]. HPyV6 and HPyV7 identified in skin and eyebrow hairs, have in one study been isolated from malignant and non-malignant tonsils [6, 38, 40–42]. HPyV9 was identified in 2010 in the serum of a kidney transplant patient under immunosuppressive treatment [8]. Its DNA was found in serum, PBMCs and skin [38, 43, 44]. HPyV10 was originally detected in stool, while the isolate MWPyV was first encountered in the skin of a patient with the “warts, hypogammaglobulinaemia, infections and myelokathexis” (WHIM) syndrome. MXPyV, another HPyV10 isolate, was found in stool and in respiratory samples, [9, 10, 45] while STLPyV DNA was amplified in stool and urine [13]. A STLPyV variant sharing 92% genome identity with the originally described MA138 and WD972 strains, was amplified from skin warts of a patient suffering from WHIM syndrome [46]. HPyV12 was found in organs of the digestive tract, particularly in the liver but also in colon, rectum and stool [47]. NJPyV DNA sequences and virions were originally detected in a muscle biopsy of a pancreatic transplant recipient with viral sequences also found in endothelial cells in muscle and skin [48].

To demonstrate clinical correlates and disease associations for HPyVs, as with most other viruses, the diagnostic cornerstones are nucleic acid detection and serodiagnosis. To this end, we have developed and validated several PCR protocols [37, 49–51]. The Luminex technology offers a novel platform for sensitive and specific, high-throughput multiplex DNA detection. An assay has earlier been set up for the detection of 10 HPyVs [52, 53]. We here describe the further development of this multiplex nucleic acid assay for the detection of all 13 HPyVs currently known, in a clinically applicable format. By combining multiplex PCR amplification with bead based hybridization and flow cytometric analysis, the resulting Luminex-based multiplex assay can simultaneously identify all the 13 HPyVs in a single reaction.

Herein, the multiplex assay is evaluated for specificity, sensitivity and reproducibility. Furthermore, we aimed to determine to what extent the lymphoid system plays a role in HPyV infection and persistence by exploring the frequencies of occurrence of these viral genomes in tonsillar biopsies from children and adults with tonsillar disease.

## Methods

### Clinical specimens

**Fig. 1:**
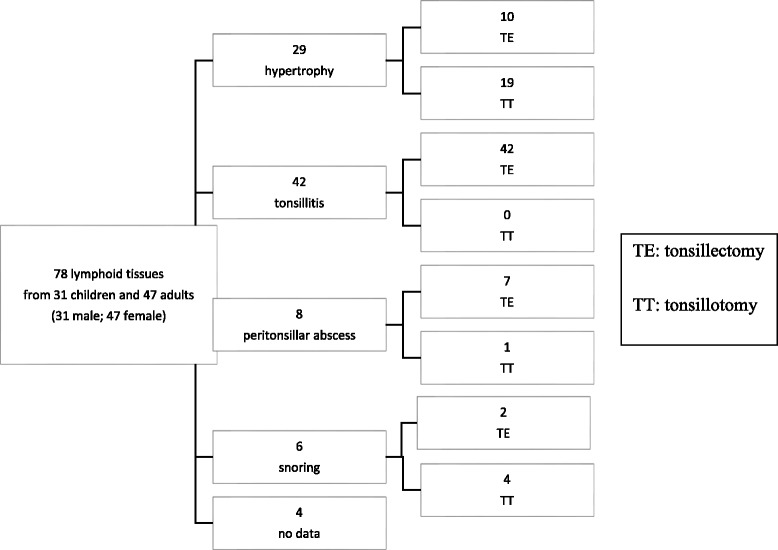
Tonsillar samples from children and adults; clinical indications

The clinical material comprised tonsillar tissue from 78 subjects: 31 children and 47 adults (Fig. 1). The pediatric donors ranged in age from 2 to 15 years (average, 6.6), and the adults from 16 to 69 (average, 30.3). Of the specimens, 31 were from males and 47 from females (Fig. 1). The tonsillectomies and tonsillotomies were performed in most cases due to chronic tonsillitis or tonsillar hypertrophy (Fig. 1). All tissues were collected and used in accordance with the ethical rules of the Ethics Committee of the Hospital District of Helsinki and Uusimaa. All tonsil tissues were freshly obtained directly after surgical resection at the operation theatre. The tonsils were cut with disposable scalpels and cell suspensions were prepared by mechanical homogenisation with a syringe plunge, followed by a wash with PBS and filtration through a 70 μm mesh (Corning Life Sciences). The cells were resuspended into final volume of 100 μL PBS.

### Nucleic acid extraction

Whole DNA was extracted from cell suspension by the KingFisher Duo DNA Extraction Kit (Thermo Fisher Scientific) according to the manufacturer’s instructions. Standard precautions to avoid contamination were taken. The extracted DNA was stored at −20 °C.

### Primer pairs and probes

Primer pairs and probes for 13 HPyVs (BKPyV, JCPyV, KIPyV, WUPyV, MCPyV, HPyV6, HPyV7, TSPyV, HPyV9, HPyV10 (MWPyV), STLPyV, HPyV12 and NJPyV), were based on GenBank sequences (Table 1). The primers and probes for the first 10 except MCPyV were the same as published by Gustafsson et al. [53] while the primers and probes for STLPyV, HPyV12 and NJPyV were designed for the present study. Primers for MCPyV were reselected due to dimerization of the published primers with primers for STLPyV. The primers were designed in current study by using the Amplify 3X software, version 3.1.4 (Bill Engels, University of Wisconsin) and assessed using an online NCBI Blast analysis. The 5′ ends of the reverse primers of the viral protein 1 (VP1) region were labeled with biotin (5′ biotinylation). The probes were 5′ amine-C12-modified (5′ Aminolink C12).

**Table 1:** Sequences of primers and probes, amplicon sizes, amplicon positions (in VP1 region) in target genome and reference strain used in the 13-plex Luminex assay [59]

Virus	Forward/Reverse (5′ biotinylated) Primers sequence (5′-3′) Probe (5′ amine-C12) sequence (5′-3′)	Amplicon length (bp)	Amplicon position (bp) in target genome	Reference strain (GenBank accession no.)
JCPyV	AATGAGGATCTAACCTGTGGAA/CTGCACCATTGTCATGAGTTGCTTG ATGAATGTGCACTCTAATGG	127	1742–1868	J02226
BKPyV	ACAGAGGTTATTGGAATAACTAG/ACTCCCTGCATTTCCAAGGG CTTAACCTTCATGCAGGGTC	143	1952–2094	DQ305492
KIPyV	TTGGATGAAAATGGCATTGG/TAACCCTTCTTTGTCTAAAATGTAGCC CTTGGAACAGCTAATAGTAGAATC	142	2263–2404	EF127906
WUPyV	TTGGATGAAAATGGCATTGG/TAACCCTTCTTTGTCTAAAATGTAGCC GAGTACATACAGGGCTTTCCAG	142	2411–2552	EF444554
MCPyV	TTCCATCTTTATCTAATTTTGCTT/AGGCCTAGTTTTAGATTACCAGAC GTAATAGGCCCACCATTTGT	144	3757–3900	EU375803
HPyV6	TTGCTTCTGGATCCAATACTGC**/**GGCCTCAGGAATTTCAGGCAA TGGATGCTGGTTCATCTCTG	131	1426–1556	HM011558
HPyV7	AAGCAGCTACAACTGGGAACTT/GGCCTCAGGAATTTCAGGCAA GCCTACCTTATCCTATGAGTG	125	1450–1574	HM011566
TSPyV	AGAATGTATGATGACAAAGGTAT/TCTGTAGTTTCCAGTTAGAAAC TGAGGGAATGAATTTCCATATGTT	111	1722–1832	GU989205
HPyV9	ATCTATGGCTCATCCTCAGG/GTAGAGCTAGCAACTAGGCCT AGTGCAGGGTACCACTCTC	107	1862–1968	KC831440
HPyV10	GTCCAGTTCCTACTAAAGTTCCT/TACATCATTGCCCATCCTTGGTT GCCGGACACCACAATGACA	106	1501–1628	JQ898292
STPyV	TGAATATGATCCGTGCCAAA/ACTGCATCAGGGCCTACTTG CCTCCTCCAACATGTGTTCC	129	1318–1446	JX463184
HPyV12	GTAATGGCACCCAAGAGGAA/GGGGATTTAGAAAGGCCTCA CCCAGCAGTGTCCCTAAATT	157	1402–1558	JX308829
NJPyV	TGTGTGCCAAAGAAGTGTCCT/TCTGTCACCTGTTGGAGCATT CTGATGCTACTACTGAAATTGAA	159	1113–1271	KF954417

Sequences for primers and probes for all except MCPyV, STPyV, HPyV12 and NJPyV as in Gustafsson et al [53].

### Plasmid clones

For use as positive controls and to determine assay sensitivities by limiting dilution analysis, plasmids containing each HPyV genome were used. Plasmid DNA was purified using the High Pure Plasmid Isolation Kit (Roche Diagnostics). The concentration of the purified plasmid DNA was determined with the NanoDrop 2000 (Invitrogen, Carlsbad, CA, USA) and the corresponding genome copies were calculated based on the concentration and molecular weight of the plasmid. A 10-fold dilution series of 10^8^ to 10^0^ copies/μL of HPyV DNA was prepared for each of the 13 HPyVs in PCR-grade H_2_O, aliquoted, and stored at −20 °C.

### Multiplex PCR for HPyV detection using the Luminex platform

All the HPyV assays were at first performed in singleplex format and then multiplexed. After confirming that each individual primer pair amplified specifically its plasmid sequence, the primer pairs were combined. The multiplex nucleic acid amplification was conducted as below. In brief, 5 μL DNA templates were mixed in a 20 μL multiplex reaction consisting of 12.5 μL of 2× multiplex PCR mastermix (Qiagen), 0.2 μM of each forward primer and 1 μM of each biotinylated reverse primer. The amplification conditions were 95 °C for 15 min, 40 cycles at 94 °C for 20 s, 50 °C for 90 s, 71 °C for 1 min and 20 s, and a final extension at 71 °C for 10 min.

### HPyV singleplex nested PCR

Each positive specimen was amplified and confirmed with the corresponding HPyV singleplex nested PCR (JCPyV, WUPyV, MCPyV, HPyV, and TSPyV). PCR-grade H_2_O was included in each experiment. In brief, 3 μL of amplified DNA template was mixed in a 22 μL multiplex reaction consisting of 12.5 μL of 2× multiplex PCR mastermix (Qiagen), 0.2 μM of the corresponding forward primer and 0.2 μM of the corresponding biotinylated reverse primer. The amplification conditions were the same as with the multiplex PCR.

### Luminex-based suspension array procedure (oligonucleotide coupling, hybridization, and measurement)

*Oligonucleotide coupling;* thirteen capture probes were included in the multiplex assay (Table 1). Each probe sequence represented the reverse complement to the target region of the biotinylated PCR product. Different sets of carboxylated fluorescent microbeads were obtained from Luminex Corp. (‘s-Hertogenbosch, The Netherlands), and oligonucleotide probes for target viruses were assigned to individual bead sets. The oligonucleotide coupling was done according to the manufacturer’s instructions (xMAP cookbook, Luminex). The probe-coupled beads were counted using a hemocytometer and were stored in the dark at +4 °C. *Hybridization;* probe-beads and PCR products were hybridized as published [53] except that the streptavidin-phycoerythrin (SAPE, Invitrogen) incubation temperature was 48 °C. After three washes the amplicons were labeled with 4 μg/mL SAPE conjugate in 2 M tetramethylammonium chloride (Sigma), 75 mM Tris, 6 mM EDTA and 1.5 g7 L sarkosyl (Sigma), pH 8.0; for 20 min in the dark. *Measurement*; after three washes, the beads and the SAPE signal were analyzed in a Bio-Plex 200 (Bio-Rad).

### Data analysis and cutoff definition

The results were measured and calculated by the software Xponent 2.1. The data were expressed as Median Fluorescence Intensity (MFI) of 100 microspheres of each bead type. The cut-off value for a positive result was defined as net MFI two times background mean plus 15 MFI.

### Specificity of HPyV multiplex assay

To evaluate the specificity of the multiplex assay, plasmids of each HPyV alone (at 10^5^ copies/μL) and combined were used as template to hybridize with a mixture of 13 type-specific probe-bead sets. Specificity of the multiplex platform was also investigated with virus-free SF9 and HEK 293 cells.

### Detection limits of HPyV multiplex assay

To determine the sensitivity of the single and multiplex assays, we tested each virus with 10-fold serial dilutions of the DNA standards. The detection limit was defined as the dilution containing the fewest copies of viral genome (in duplicate testing) that still gave a positive result.

### Reproducibility of HPyV multiplex assay

To test the multiplex assay reproducibility, a mixture of all 13 HPyV plasmids (at 10^3^ copies/μL/each HPyV) was analyzed with a mixture of 13 type-specific probe-bead sets. Intra-assay variation was calculated with triplicates in the same run, and inter-assay variation in 3 distinct runs.

## Results

We designed a multiplex assay for the detection and identification of 13 HPyVs, by extension of a previously designed multiplex assay for 10 HPyVs [53]. The assay conditions in this study were optimized for the following parameters: amounts of HPyV-specific probes, each coupled to a distinct Luminex bead, amounts of forward primer and biotinylated reverse primer; hybridization conditions; SAPE concentration; incubation time for SAPE staining; and numbers of washing cycles (data not shown).

### Specificity of HPyV multiplex assay

PCR-grade H_2_O was included in each experiment to determine background. Specificity of the assay was assessed with plasmids containing the respective HPyV VP1 inserts. Plasmids of each of the 13 HPyVs alone and combined (10^5^ copies/μl/each HPyV) were used as template for singleplex/multiplex amplification and hybridization. The multiplex assay did identify correctly all the 13 target sequences, with no cross reactions (Table 2). Furthermore, we tested the specificity of the multiplex assay with virus-free SF9 and HEK 293 cells, with no positive signals obtained (data not shown).

**Table 2:** Specificities of 13 type-specific probes employed in multiplex HPyV genotyping

Target sequence	HPyV type-specific probe (MFI)												
	BKPyV	JCPyV	KIPyV	WUPyV	MCPyV	HPyV6	HPyV7	TSPyV	HPyV9	HPyV10	HPyV11	HPyV12	HPyV13
BKPyV	2105	43	35	36	33	54	42	27	34	34	44	35	19
JCPyV	39	1145	32	32	32	34	34	24	39	28	47	32	20
KIPyV	49	48	2522	45	42	43	43	38	48	44	59	47	17
WUPyV	33	36	29	5704	31	31	65	18	35	26	43	29	19
MCPyV	46	46	37	38	2736	45	41	31	40	37	49	39	21
HPyV6	38	38	34	32	33	3196	35	30	34	31	44	35	23
HPyV7	43	46	38	52	35	42	2574	29	41	41	51	37	18
TSPyV	52	52	45	74	42	46	64	2894	47	42	58	47	19
HPyV9	50	48	44	40	47	47	39	29	1388	39	54	49	18
HPyV10	38	36	33	32	34	36	32	21	33	5116	47	31	24
HPyV11	47	56	42	51	45	66	61	57	59	58	1782	66	17
HPyV12	55	50	42	48	45	50	46	38	46	39	61	681	20
HPyV13	39	42	36	36	37	39	40	27	40	35	48	37	2327
Mix HPyVs	745	1367	1518	2884	1511	1586	1125	733	603	2639	612	326	1631
cutoff	85	81	77	81	77	83	77	75	81	72	107	73	127

Each line represents a single well with each HPyV plasmid (10^5^ copies/μL) or plasmid mix hybridized to a mixture of 13 distinct beads

### Limit of detection of HPyV multiplex assay

To determine the sensitivities of the single- and multiplex assays, each HPyV plasmid was diluted serially from 10^8^ to 10^0^ in PCR-grade H_2_O. The limit of detection was the dilution containing the fewest copies in duplicate of viral genome that still gave a positive result. The analytical sensitivities in singleplex and multiplex format ranged from 10^0^ to 10^2^ copies per microliter with all 13 HPyVs (Table 3).

**Table 3:** Limits of detection of 13 HPyVs in singleplex and multiplex format

HPyV	Limits of detection per μL of DNA extract	
	Singleplex	Multiplex
BKPyV	10^1^	10^1^
JCPyV	10^1^	10^1^
KIPyV	10^1^	10^1^
WUPyV	10^1^	10^1^
MCPyV	10^1^	10^2^
HPyV6	10^1^	10^1^
HPyV7	10^2^	10^2^
TSPyV	10^1^	10^1^
HPyV9	10^1^	10^2^
HPyV10	10^0^	10^1^
HPyV11	10^1^	10^2^
HPyV12	10^0^	10^1^
HPyV13	10^1^	10^1^

### Intra-assay and inter-assay reproducibility of HPyV multiplex assay

The reproducibility of HPyV multiplex assay was determined with a mixture of all 13 HPyV plasmids (10^3^ copies/μL/each HPyV) and a mixture of 13 type-specific probe-bead sets. The coefficient of variation obtained from triplicates in a single run ranged from 2.9% to 9.2% and from three independent runs ranged from 5.7% to 18.8%.

### Application of HPyV multiplex assay on tissues

The multiplex assay was applied to tonsillar samples obtained from children and adults. Among the 78 tissue donors the assay tested positive as follows: BKPyV (*n* = 0), JCPyV (*n* = 1), KIPyV (*n* = 0), WUPyV (*n* = 3), MCPyV (*n* = 1), HPyV6 (*n* = 6), HPyV7 (*n* = 0), TSPyV (*n* = 3), HPyV9 (*n* = 0), and HPyV10 (*n* = 0), STLPyV (*n* = 0), HPyV12 (*n* = 0), and NJPyV (*n* = 0). Co-infection of WUPyV and TSPyV was observed in one tissue. Altogether 13 specimens tested positive: JCPyV DNA was found in a female (22 year old) with tonsillitis and peritonsillar abscess; MCPyV DNA in a female (46 y) with tonsillitis; and HPyV6 DNA in 4 males and 2 females (33 y median age [range, 14 to 69]) with diverse tonsillar conditions. WUPyV DNA occurred in two children (male, aged <5 years; one with snoring and the other with hypertrophy). TSPyV DNA was detected in a 2-y child (male) with hypertrophy and an 11-y child (male) with no clinical data available. Co-infection with WUPyV and TSPyV was seen in a 6-y child (female) with hypertrophy. Each positive specimen was confirmed with the corresponding PyV singleplex nested PCR(s). No product was generated from the negative control.

## Discussion

The number of HPyVs known has multiplied explosively during the past decade. Infections by these viruses appear ubiquitous since birth. Their DNAs have been found widely in the human body, including lymphoid organs, albeit in low copy numbers [54–60]. Whereas JCPyV, BKPyV, MCPyV and TSPyV are known to be associated with diseases, the others are orphan as regards clinical manifestations.

The currently existing PCR assays are of limited value in multi-HPyV assessment. To this end, sensitive detection methods covering all the HPyVs are needed. By extension and optimization of an earlier one [53], we here describe the development and validation of a Luminex-based multiplex assay that allows for simultaneous detection of 13 HPyVs. The new assay exhibited a high analytical sensitivity, i.e., ability to detect all HPyVs with detection limits of 10^0^–10^2^ copies per microliter, and suitability for high-throughput analysis. We consider the method advantageous also for analyzing multiple infections. This was demonstrated by the detection of up to 13 types in mixes of HPyV plasmids, and of 2 HPyVs in a single tonsillar sample.

In a previous study [61] addressing the detection of members of *Polyomaviride* in tonsillar tissues from Chinese children with chronic tonsillar disease, WUPyV, MCPyV, TSPyV, KIPyV, MWPyV, and STLPyV were detected in eleven (11%), four (4.0%), three (3.0%), two (2.0%), two (2.0%), and two (2.0%) of 99 samples, respectively. A single HPyV infection was found in 24 (24%) of the samples, and dual HPyV infections (WUPyV and MCPyV) in 2 (2%) samples. In our previous study, we investigated 229 matched pairs of tonsillar tissue biopsies and corresponding serum samples for the presence of TSPyV and found this emerging HPyV in 8 (3.5%) tonsils, and in none of the sera [37]. Each of the 8 PCR-positive subjects had antiviral IgG of high avidity but not IgM, disclosing persistence. In the present study, we detected JCPyV, WUPyV, MCPyV, HPyV6, and TSPyV DNA altogether in 14 (18%) of 78 tonsils, and co-infection of two HPyVs (WUPyV and TSPyV) in one tissue. All of these data provide evidence of tonsils serving as shedding site in HPyV reactivation and thus contributing to HPyV transmission.

### Note added in submission

After completion of our work, Herberhold et al. (Med Microbiol Immunol, DOI 10.1007/s00430%E2%80%93016%E2%80%930486-6) published tonsillar HPyV DNA prevalence data highly similar to ours by using real-time quantitative PCRs in singleplex format.

## Conclusion

A Luminex-based multiplex assay was developed for epidemiological and diagnostic studies to address whether any of the emerging HPyVs, or infection with HPyV thereof, is associated with disease development. Based on the observed frequent occurrence of HPyVs in human tonsils, we suggest that lymphoid tissue may be a general persistence site for these viruses. Thereby, shedding from this site during reactivation might play a role in HPyV transmission.

## Abbreviations

BBMA: Bead-based multiplex assay
BKPyV: BK polyomavirus
HPyVs: Human polyomaviruses
JCPyV: JC polyomavirus
MCC: Merkel cell carcinoma
MCPyV: Merkel cell polyomavirus
MFI: Median fluorescence intensity
NJPyV: New Jersey polyomavirus
SAPE: Streptavidin-phycoerythrin
STLPyV: Saint Louis polyomavirus
TS: Trichodysplasia spinulosa
TSPyV: Trichodysplasia spinulosa
VP1: Viral protein 1
WHIM: Warts, hypogammaglobulinaemia, infections and myelokathexis
